# Supplementary material for: Comparative Analysis of Aristolochic Acids in Aristolochia Medicinal Herbs and Evaluation of Their Toxicities
Source: Toxins (Basel). 2022 Dec 16;14(12):879. doi: 10.3390/toxins14120879 (PMC9783648; doi:10.3390/toxins14120879)
Supplement: Supplementary file 1 [file toxins-14-00879-s001.zip › toxins-2052805-supplementary.pdf]

Supporting Information

Comparative analysis of aristolochic acids in *Aristolochia* medicinal herbs and evaluation of their toxicities

Content

Figure S1 MS/MS fragments of AAI, AAII, AAD, Al-I, and AL-II. 3

Figure S2 Typical LC-MS/MS total ion chromatograms (TIC) of the whole herb of AMH in positive ion modes. 4

Table S1 Constituents identified information of AMH....4

Figure S3 Typical LC-MS/MS total ion chromatograms (TIC) of the whole herb of ADS in positive ion modes. 7

Table S2 Constituents identified information of ADS....7

Figure S4 Typical LC-MS/MS total ion chromatograms (TIC) of the whole herb of ACY in positive ion modes. 10

Table S3 AAs component identification information for ACY 10

Table S4 Sample information of AMH....13

Table S5 Sample information of ADS....14

Table S6 Sample information of ACY....15

Figure S5 Results of comet assay 16

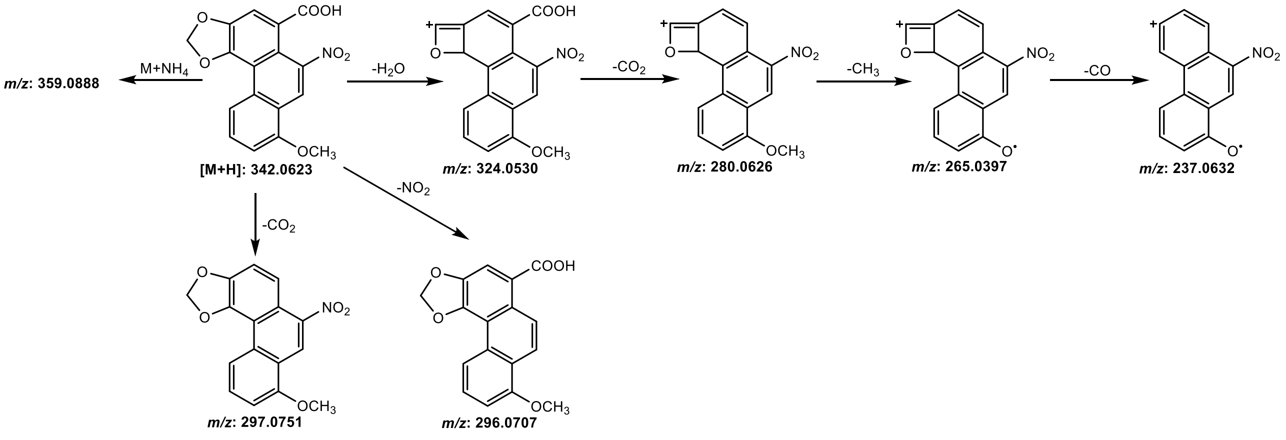

A

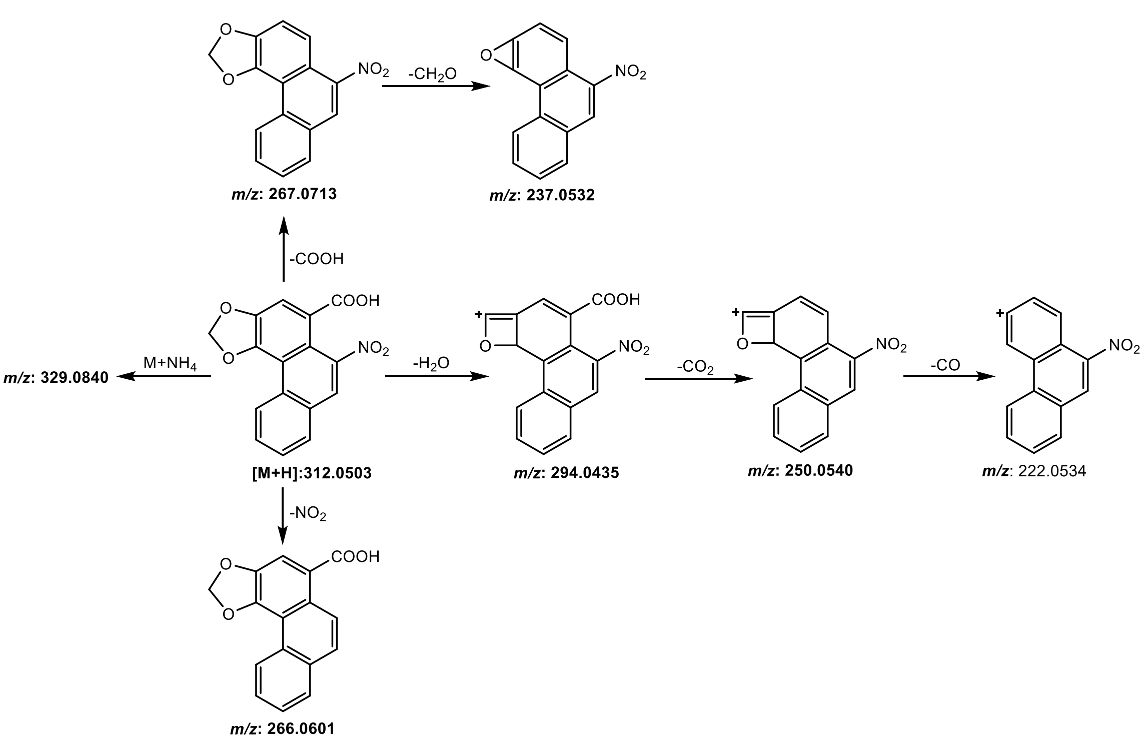

NO2
-CH2O
O
NO2
m/z: 267.0713
m/z: 237.0532
-COOH
COOH
NO2
M+NH4
m/z: 329.0840
-H2O
COOH
NO2
-CO2
NO2
-CO
NO2
[M+H]:312.0503
m/z: 294.0435
m/z: 250.0540
m/z: 222.0534
-NO2
COOH
m/z: 266.0601

B

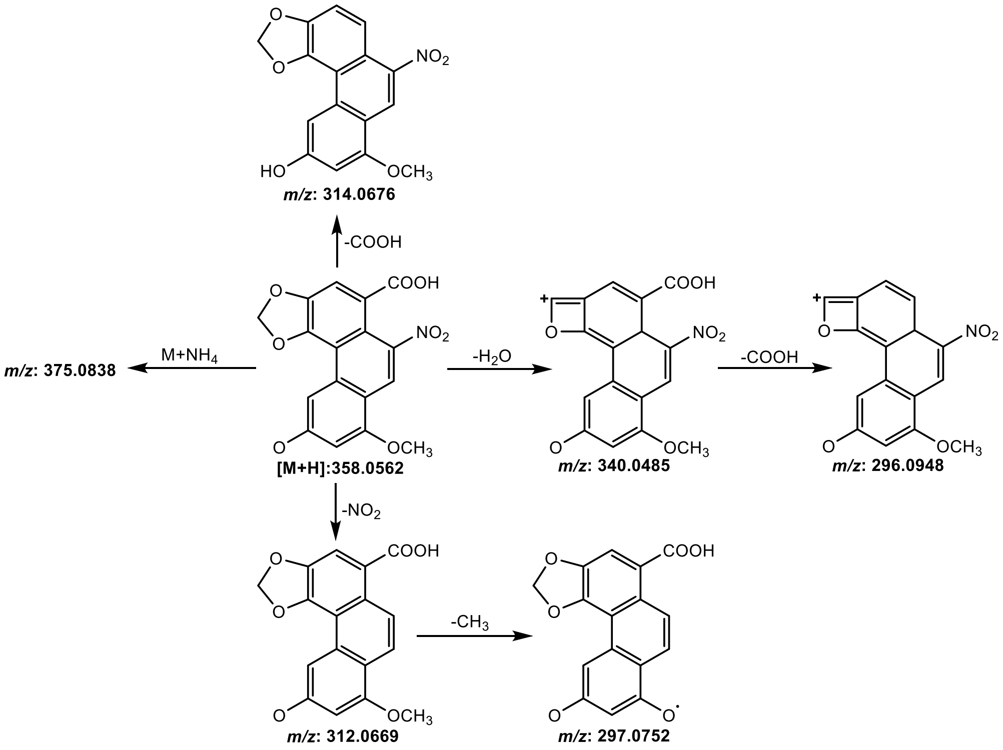

NO2
HO
OCH3
m/z: 314.0676
-COOH
COOH
NO2
M+NH4
m/z: 375.0838
-H2O
COOH
NO2
-COOH
NO2
OCH3
[M+H]:358.0562
m/z: 340.0485
m/z: 296.0948
-NO2
COOH
-CH3
COOH
OCH3
m/z: 312.0669
m/z: 297.0752

C

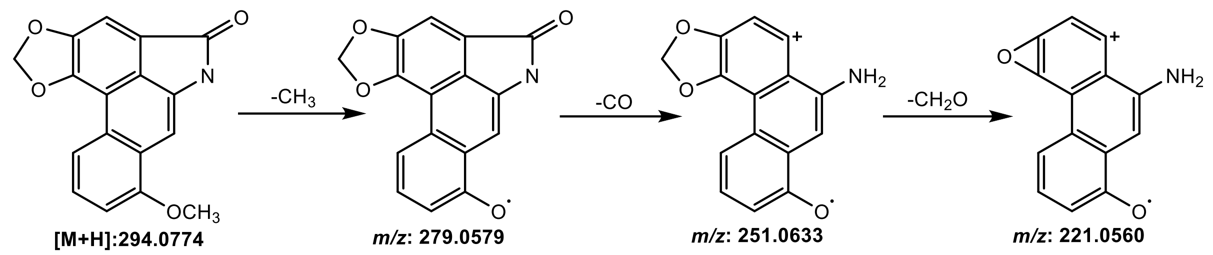

D

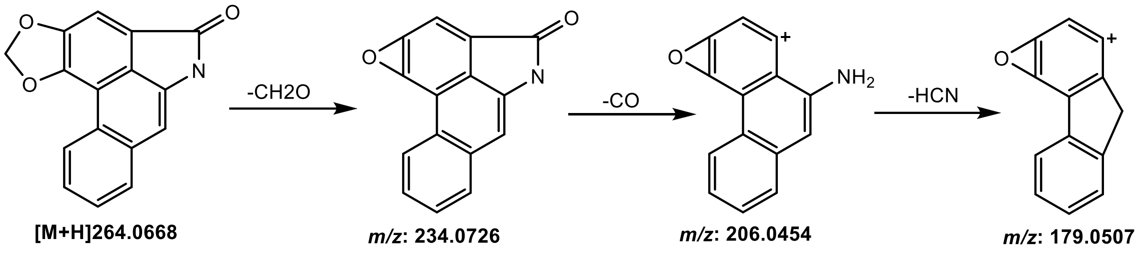

E

A.AAI; B.AAII; C.AAD; D.AL-I; E.AL-II

Figure S1. MS/MS fragments of AAI, AAII, AAD, Al-I, and AL-II.

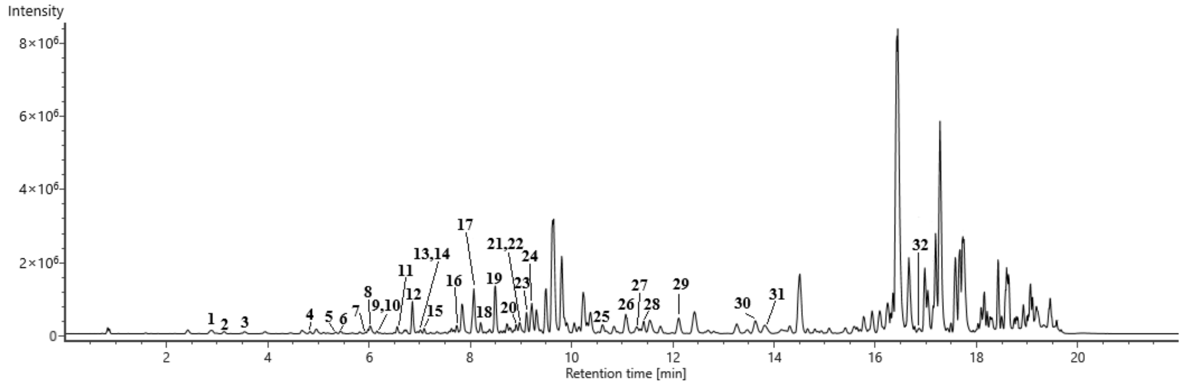

Figure S2 Typical LC-MS/MS total ion chromatograms (TIC) of the whole herb of AMH in positive ion modes.

Table S1 Constituents identified information of AMH

Peak number	AssAssigned **identity**	Molecular formula	Theoretical exact mass (Da) [M + H]+	Positive ion mode			MS/MS fragments
				tR (min)	Mean measured mass (Da) [M + H]+	Mass accuracy (ppm)	
1	cuminyl alcohol	$C_{10}H_{14}O$	151.1117	3.02	151.1113	-2.65	133.0994 105.0684 91.0527
2	SALICYLAMIDE	$C_7H_7NO_2$	138.0549	3.27	138.0541	-5.79	121.0267 93.0316 77.0376
3	trans-Ferulic acid	$C_{10}H_{10}O_4$	195.0651	3.31	195.0659	4.10	149.0613 134.0723 117.0334 84.9274
4	ferulic acid	$C_{10}H_{10}O_4$	195.0651	4.86	195.065	-0.51	145.0101 135.0103 117.0009 89.0065
5	2-(1,2-dihydroxyethyl)-3-[(E)-3-(3,4-dihydroxyphenyl)prop-2-enoyl]oxy-4-hydroxy-3,4-dihydro-2H-pyran-6-carboxylic acid	$C_{17}H_{18}O_{10}$	383.0972	5.31	383.0957	-3.92	266.0975 163.0367 135.0418 117.0328
6	N-p-trans-Coumaroyltyramine	$C_{17}H_{17}NO_3$	284.1281	5.83	284.1269	-4.22	147.0110 122.0634 91.0223
7	Quercetin-4'-O-glucoside	$C_{21}H_{20}O_{12}$	465.1027	5.98	465.1047	4.30	303.0242 246.0652
8	Rutin	$C_{27}H_{30}O_{16}$	611.1606	6.17	611.1632	4.25	465.0923 449.0951 303.0248 229.0175
9	6,7-dihydroxychromen-2-one	$C_9H_6O_4$	179.0338	6.25	179.0336	-1.12	151.0405 135.0497 133.0332 123.0339
10	Spiraeoside	$C_{21}H_{20}O_{12}$	465.1027	6.27	465.1042	3.23	449.0951 303.0248 325.0345
11	Quercetin 3-O-alpha-rhamnopy ranoside	$C_{21}H_{20}O_{11}$	449.1078	6.54	449.1059	-4.32	433.1012 177.0215 287.0273 147.0088
12	3-[(2S,3R,4S,5S,6R)-4,5-dihydroxy-6-(hydroxymethyl)-3-[(2S,3R,4S,5R)-3,4,5-trihydroxyoxan-2-	$C_{27}H_{30}O_{16}$	611.1606	6.83	611.1616	1.64	479.1108 317.0405 169.0874

	yl]oxyoxan-2-yl]oxy-2-(3,4-dihydroxyphenyl)-5-hydroxy-7-methoxychromen-4-one						
13	Isorhamsin-3-O-galactoside	$C_{22}H_{22}O_{12}$	479.1184	6.93	479.1169	-3.13	317.0412 302.0152 246.1195 163.0046
14	4-(3,4-dihydroxyphenyl)-7-hydroxy-5-[3,4,5-trihydroxy-6-[(3,4,5-trihydroxyoxan-2-yl)oxymethyl]oxan-2-yl]oxychromen-2-one	$C_{26}H_{28}O_{15}$	581.1501	6.99	581.1497	-0.69	449.1078 419.0983 287.0535 251.1052
15	2-(3,4-dihydroxyphenyl)-5-hydroxy-7-(((2S,3R,4S,5S,6R)-3,4,5-trihydroxy-6-((((2R,3R,4R,5R,6S)-3,4,5-trihydroxy-6-methyltetrahydro-2H-pyran-2-yl)oxy)methyl)tetrahydro-2H-pyran-2-yl)oxy)-4H-chromen-4-one	$C_{27}H_{30}O_{15}$	595.1657	7.12	595.1685	4.62	449.1071 287.0546
16	Drospirenone	$C_{24}H_{30}O_3$	367.2267	7.74	367.2282	4.08	220.1678 163.0371
17	Tiliroside	$C_{30}H_{26}O_{13}$	595.1446	8.06	595.147	4.03	565.1556 309.0996 287.0547 147.0430
18	3-[(2S,3R,4S,5R,6R)-6-[[(2R,3R,4R,5S,6S)-3,5-dihydroxy-6-methyl-4-[(2S,3R,4R,5R,6S)-3,4,5-trihydroxy-6-methyloxan-2-yl]oxyoxan-2-yl]oxymethyl]-3,4,5-trihydroxyoxan-2-yl]oxy-5,7-dihydroxy-2-(4-hydroxyphenyl)chromen-4-one	$C_{33}H_{40}O_{19}$	741.2236	8.12	741.2227	-1.21	565.1556 472.1228 287.0568
19	(E)-3-(4-hydroxy-3-methoxyphenyl)-N-[2-(4-hydroxyphenyl)ethyl]prop-2-enamide	$C_{18}H_{19}NO_4$	314.1386	8.48	314.1374	-4.07	177.0220 145.0684 121.0305
20	[6-[2-(3,4-dihydroxyphenyl)-8-hydroxy-4-oxochromen-7-yl]oxy-3,4,5-trihydroxyoxan-2-	$C_{30}H_{26}O_{13}$	595.1446	8.90	595.1462	2.69	288.0935 147.0869 153.0208

	yl]methyl (E)-3-(4-hydroxyphenyl)prop-2-enoate						
21	Cyanidin 3-rutinoside	$C_{27}H_{31}ClO_{15}$	631.1424	8.96	631.1414	-1.62	613.1414 287.0512
22	[5-hydroxy-6-[2-(4-hydroxy-3-methoxyphenyl)ethoxy]-2-(hydroxymethyl)-4-(3,4,5-trihydroxy-6-methyloxan-2-yl)oxyoxan-3-yl] (E)-3-(4-hydroxy-3-methoxyphenyl)prop-2-enoate	$C_{31}H_{40}O_{15}$	653.2440	8.96	653.2418	-3.37	177.0534 145.0097
23	kaempferol	$C_{15}H_{10}O_{6}$	287.0550	9.18	287.0544	-2.13	233.1520 153.0171
24	2-(3,4-dihydroxyphenyl)-3,5,7-trihydroxychromen-4-one	$C_{15}H_{10}O_{7}$	303.0499	9.29	303.0487	-4.06	228.0896 203.0627 185.0539
25	1-methyl-4-methylidene-7-(propan-2-yl)-1,2,3,3a,4,5,6,8a-octahydroazulen-1-ol	$C_{15}H_{26}O$	223.2056	10.64	223.2067	4.93	161.1308 133.0999 119.0838 105.0688 91.0530
26	luteolin 4'-O-glucoside	$C_{21}H_{20}O_{11}$	449.1078	11.11	449.1086	1.78	287.0276 153.0199
27	13S-Hydroxy-9Z,11E,15Z-octadecatrienoic acid	$C_{18}H_{30}O_{3}$	295.2267	11.31	295.2258	-3.05	277.2147 259.2048 135.1154
28	Aurantiamide acetate	$C_{28}H_{30}N_{2}O_{4}$	459.2278	11.47	459.2298	4.36	203.1426 105.0688 145.1001 91.0591
29	2-(3,4-dihydroxyphenyl)-3-[(2S,3R,4S,5S,6R)-4,5-dihydroxy-3-[(2R,3R,4R,5R,6S)-3,4,5-trihydroxy-6-methyloxan-2-yl]oxy-6-[[(2R,3R,4R,5R,6S)-3,4,5-trihydroxy-6-methyloxan-2-yl]oxymethyl]oxan-2-yl]oxy-5,7-dihydroxychromen-4-one	$C_{33}H_{40}O_{20}$	757.2185	12.21	757.2197	1.58	427.2676 303.0483
30	Homoorientin	$C_{21}H_{20}O_{11}$	449.1078	13.62	449.1065	-2.89	431.1050 395.0659 383.1136 353.0210
31	2-(3,4-Dihydroxyphenyl)-5,7-dihydroxy-8-[2-O-[(2E)-3-(4-hydroxyphenyl)-1-oxo-2-propen-1-yl]-beta-D-glucopyranosyl]-4H-1-benzopyran-4-one	$C_{30}H_{26}O_{13}$	595.1446	13.85	595.1467	3.53	289.0162 147.0473
32	Isorhamnetin	$C_{16}H_{12}O_{7}$	317.0655	16.92	317.0645	-3.15	285.0537 275.0705

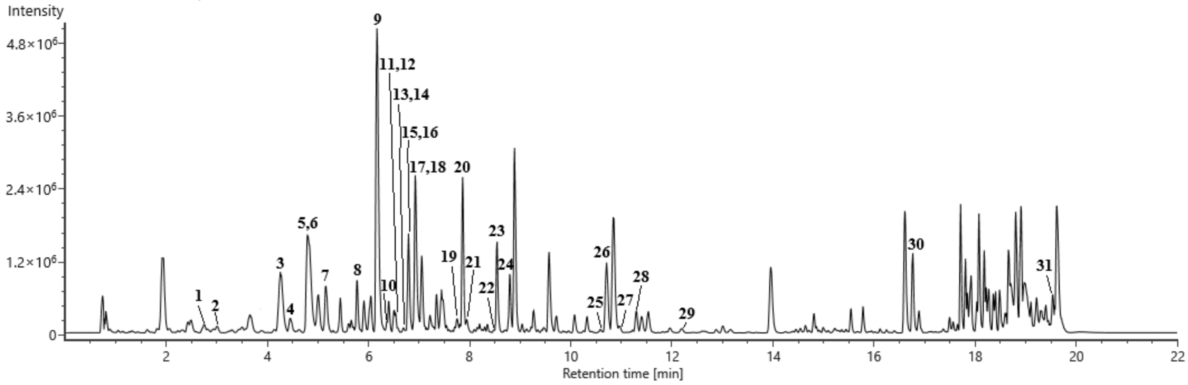

Figure S3 Typical LC-MS/MS total ion chromatograms (TIC) of the whole herb of ADS in positive ion modes.

Table S2 Constituents identified information of ADS

Peak number	Assigned identity	Molecular formula	Theoretical exact mass (Da) [M + H]+	Positive ion mode			MS/MS fragments
				tR (min)	Mean measured mass (Da) [M + H]+	Mass accuracy (ppm)	
1	4-[(E)-3-(3,4-dihydroxyphenyl)prop-2-enoyl]oxy-2,3-dihydroxy-2-methylbutanoic acid	$C_{14}H_{16}O_8$	313.0917	2.79	313.0907	-3.19	163.0053 144.9953 135.0136 117.0035
2	cuminyl alcohol	$C_{10}H_{14}O$	151.1117	3.02	151.1121	2.65	133.1374 131.1210 105.1054 91.0882
3	3-Hydroxy-7-methoxyflavone	$C_{16}H_{12}O_4$	269.0808	4.26	269.0807	-0.37	237.0620 209.0644 107.0166
4	(1R)-(-)-Nopol	$C_{11}H_{18}O$	167.143	4.48	167.1429	-0.60	149.1298 121.984 107.0834
5	ferulic acid	$C_{10}H_{10}O_4$	195.0651	4.86	195.0660	4.61	145.0101 135.0103 117.0009 89.0065
6	caffeic acid	$C_9H_8O_4$	181.0495	4.88	181.0492	-1.66	144.9955 135.0108 117.0009
7	5-hydroxy-2-[1-hydroxy-4-[(2R,3R,4S,5S,6R)-3,4,5-trihydroxy-6-	$C_{22}H_{24}O_{11}$	465.1391	5.16	465.1403	2.49	445.1611 309.0722 291.0613 242.0344 147.0166

	(hydroxymethyl)oxan-2-yl]oxycyclohexa-2,5-dien-1-yl]-7-methoxychromen-4-one						
8	N-p-trans-Coumaroyltyramine	$C_{17}H_{17}NO_3$	284.1281	5.83	284.1282	0.35	147.0110 122.0634 91.0223
9	Rutin	$C_{27}H_{30}O_{16}$	611.1606	6.17	611.1585	-3.44	465.0923 449.0951 303.0248 229.0175
10	Spiraeoside	$C_{21}H_{20}O_{12}$	465.1027	6.37	465.1043	3.44	449.0951 303.0248 325.0345
11	Aflatoxin	$C_{21}H_{20}O_{10}$	433.1129	6.51	433.1125	-0.92	287.0294 357.0770 144.9946
12	Quercetin 3-O-alpha-rhamnopy ranoside	$C_{21}H_{20}O_{11}$	449.1078	6.54	449.1062	-3.56	433.1012 177.0215 287.0273 147.0088
13	ISOVITEXIN	$C_{21}H_{20}O_{10}$	433.1129	6.78	433.1118	-2.54	415.1007 397.09
14	2-(3,4-dihydroxyphenyl)-5-hydroxy-7-(((2S,3R,4S,5S,6R)-3,4,5-trihydroxy-6-((((2R,3R,4R,5R,6S)-3,4,5-trihydroxy-6-methyltetrahydro-2H-pyran-2-yl)oxy)methyl)tetrahydro-2H-pyran-2-yl)oxy)-4H-chromen-4-one	$C_{27}H_{30}O_{15}$	595.1657	6.79	595.1645	-2.02	449.0971 287.0277 197.0850
15	2,4,7,9-Tetramethyldec-5-in-4,7-diol	$C_{14}H_{26}O_2$	227.2005	6.85	227.1999	-2.64	191.1789 149.1367 121.1008 91.0893
16	5-hydroxy-2-(4-hydroxyphenyl)-7-((3,4,5-trihydroxy-6-methyltetrahydro-2H-pyran-2-yl)oxy)-3-((3,4,5-trihydroxytetrahydro-2H-pyran-2-yl)oxy)-4H-chromen-4-one	$C_{26}H_{28}O_{14}$	565.1551	6.86	565.1573	3.89	433.1066 287.0274 163.0062
17	Isorhamsin-3-O-galactoside	$C_{22}H_{22}O_{12}$	479.1184	6.93	479.1162	-4.59	317.0412 302.0152 246.1195 163.0046
18	5,7-dihydroxy-2-(4-hydroxy-3-methoxyphenyl)-3-[3,4,5-trihydroxy-6-[[(2R,3R,4R,5R,6S)-3,4,5-trihydroxy-6-methyloxan-2-yl]oxymethyl]oxan-2-yl]oxychromen-4-one	$C_{28}H_{32}O_{16}$	625.1763	6.93	625.1756	-1.12	479.1112 317.0412 246.1195

19	Drospirenone	$C_{24}H_{30}O_3$	367.2267	7.73	367.2258	-2.64	349.0057 333.0333
20	Parietin	$C_{16}H_{12}O_5$	285.0757	7.97	285.0763	2.10	255.0648 191.1089
21	7-hydroxy-3-(4-hydroxyphenyl)-4H-chromen-4-one	$C_{15}H_{10}O_4$	255.0651	7.99	255.0643	-3.14	225.05 223.04 195.05
22	(E)-3-(4-hydroxy-3-methoxyphenyl)-N-[2-(4-hydroxyphenyl)ethyl]prop-2-enamide	$C_{18}H_{19}NO_4$	314.1386	8.48	314.1372	-4.46	177.0220 145.0684 121.0305
23	Graveoline	$C_{17}H_{13}NO_3$	280.0968	8.57	280.0981	4.64	265.0858 163.0405
24	5-hydroxy-3-(4-methoxyphenyl)-7-((3,4,5-trihydroxy-6-(((3,4,5-trihydroxy-6-methyltetrahydro-2H-pyran-2-yl)oxy)methyl)tetrahydro-2H-pyran-2-yl)oxy)-4H-chromen-4-one	$C_{28}H_{32}O_{14}$	593.1864	8.89	593.1834	-5.06	447.1133 422.2739 323.0295 285.0489
25	sinomenine	$C_{19}H_{23}NO_4$	330.16998	10.64	330.1718	5.75	275.2004 259.2267 207.0138
26	Nandrolone	$C_{18}H_{26}O_2$	275.2005	10.76	275.2004	-0.36	257.1911 239.1810 197.1332 163.1159
27	luteolin 4'-O-glucoside	$C_{21}H_{20}O_{11}$	449.1078	11.11	449.1086	1.78	287.0276 153.0199
28	13S-Hydroxy-9Z,11E,15Z-octadecatrienoic acid	$C_{18}H_{30}O_3$	295.2267	11.30	295.2259	-2.71	277.1910 192.1060
29	2-Butanone, 4-(2,6,6-trimethyl-2-cyclohexen-1-yl)	$C_{13}H_{22}O$	195.1743	12.20	195.1757	7.17	135.1479 121.1348
30	3,5-Dimethyladamantan-1-amine	$C_{12}H_{22}ClN$	216.1513	16.88	216.1512	-0.46	107.0506 95.0530
31	9-Octadecenamide, (Z)	$C_{18}H_{35}NO$	282.2791	19.53	282.2785	-2.13	563.5550 282.2517 265.2233 247.2094

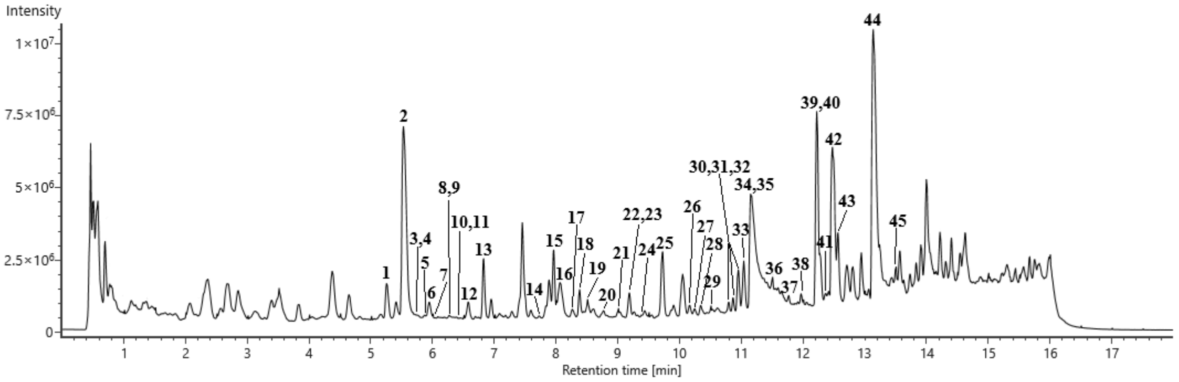

Figure S4 Typical LC-MS/MS total ion chromatograms (TIC) of the whole herb of ACY in positive ion modes.

Table S3 AAs component identification information for ACY

Peak number	Assigned identity	Molecular formula	Theoretical exact mass (Da) [M + H]+	Positive ion mode			MS/MS fragments	Adducts	relative content
				tR (min)	Mean measured mass (Da) [M + H]+	Mass accuracy (ppm)			
1	Cinnabarin	$C_{23}H_{19}NO_{10}$	470.1082	5.30	470.1091	1.91	308.0541 280.0595 252.0598	+H	0.1435
2	Aristolactam Ia-N-β-D-glucoside	$C_{22}H_{19}NO_9$	442.1133	5.58	442.1119	-3.17	424.1021 280.0595 250.0349	+H	0.1723
3	aristolic acid II-8-O-β-D-glucoside	$C_{22}H_{19}NO_{12}$	490.0980	5.70	490.0991	2.24	507.1232 328.0457 310.0351 266.0458 284.0569 254.0581 338.0440 310.0512 282.0586	+H, +NH_4	0.0057
4	Aristolactam-CIV	$C_{18}H_{15}NO_5$	326.1023	5.73	326.1019	-1.23	343.1288 311.1000 296.1593 268.1546	+H, +NH_4	0.0075
5	aristoliukine A	$C_{17}H_{13}$NO5	312.0866	5.82	312.0854	-3.85	329.11 297.0688 269.1190 239.1677	+H	0.0217
6	Aristolactam IIIa-O-β-D-glucoside	$C_{22}H_{19}NO_{12}$	490.0980	5.96	490.0959	-4.28	472.0795 444.1109 310.0356 284.0541 282.0511	+H	0.0023
7	aristolic acid I	$C_{17}H_{12}O_5$	297.0758	6.02	297.0764	2.02	279.0673 253.0874 251.1106 223.0793 221.0992	+H	0.0162
8	Aristolactam IVa-O-β-D-glucoside	$C_{23}H_{21}NO_{13}$	520.1086	6.24	520.1108	4.23	502.2957 476.1609 474.1169 340.0442 314.0908 312.0634 297.0913	+H	0.1598
9	aristchamic B	$C_{16}H_{17}NO_4$	288.1230	6.29	288.1243	4.51	255.0900	+H	0.0017

10	aristolactam A IIIa	$C_{16}H_{11}NO_4$	282.0760	6.39	282.0764	1.42	267.0766 239.0854	+H	0.0016
11	aristololide	$C_{17}H_{10}O_5$	295.0601	6.44	295.0610	3.05	312.0867 280.0923 262.1090	+H, +NH4	0.0014
12	7-hydroxyl-8-methoxyaristolate	$C_{17}H_{12}O_6$	313.0706	6.60	313.0721	4.79	*295.0604 280.0600 *269.0812 241.0468	+H	0.0076
13	Aristolactam IIIa-N-β-D-glucoside	$C_{22}H_{19}NO_9$	442.1133	6.87	442.1137	0.90	424.1039 280.0611 250.0258	+H	0.1945
14	AL-Ⅰa	$C_{16}H_9NO_4$	280.0604	7.74	280.0592	-4.28	251.0738 222.0342	+H	0.0157
15	aristoliukine C	$C_{18}H_{13}NO_5$	324.0866	7.90	324.0851	-4.63	346.0700 292.0598 280.0588 250.0513	+Na	0.0049
16	Aristolactam II-N-β-D-glucoside	$C_{22}H_{19}NO_8$	426.1183	8.02	426.1201	4.22	408.1095 264.0654 234.0559	+H	0.0049
17	Aristolochic acid III	$C_{17}H_{11}NO_7$	342.0608	8.22	342.0622	4.09	364.0438 266.0573 249.0558 237.0492	+Na	0.0075
18	Aristolactam I-N-β-D-glucoside	$C_{23}H_{21}NO_9$	456.1289	8.45	456.1291	0.44	438.1230 294.0791 279.0523	+H	0.0228
19	6-methoxydenitroaristolochic acid methyl ester	$C_{19}H_{16}O_6$	341.1019	8.58	341.1009	-2.93	358.1301 323.1381 308.1054 264.1323 249.1543 234.1590	+H, +NH4	0.0029
20	aristophyllides C	$C_{43}H_{29}NO_7$	672.2017	8.74	672.2000	-2.53	710.159 530.1122 308.0788 290.0690	+H,+K	0.0016
21	AAC	$C_{16}H_9NO_7$	328.0452	9.09	328.0464	3.66	310.0377 284.0601 282.0550	+H,+ Na	0.6858
22	aristofolin E	$C_{17}H_{12}O_4$	281.0808	9.20	281.0820	4.27	263.0946 235.0984 251.1047	+H	0.0015
23	Aristolactam III	$C_{17}H_{11}NO_4$	294.0761	9.22	294.0781	6.80	279.0523 251.0573	+H	0.0053

24	aristchamic A	$C_{19}H_{15}NO_8$	386.0870	9.38	386.0864	-1.55	368.0776	+H	0.0158
25	AL-IIIa	$C_{16}H_9NO_4$	280.0604	9.68	280.0606	0.71	250.0525 222.0387	+H	0.0130
26	aristochamic C or D	$C_{28}H_{25}NO_{11}$	552.1500	10.21	552.1515	2.72	574.1338 463.1723 443.1027 340.0862 323.0624	+Na	0.0014
27	7-OH-AAI	$C_{17}H_{11}NO_8$	358.0557	10.32	358.0567	2.79	340.0442 314.0666 312.0634	+H, +NH_4	0.1055
28	AAD	$C_{17}H_{11}NO_8$	358.0557	10.35	358.0546	-3.07	340.0445 314.0646 312.0636 297.0632 284.0625	+H, +NH_4	0.4373
29	Aristolactam AII	$C_{16}H_{11}NO_3$	266.0811	10.52	266.0812	0.38	251.0884 223.0597 195.0631 167.0700	+H	0.0045
30	aristolactam-CV	$C_{17}H_{13}NO_4$	296.0917	10.74	296.0926	3.04	281.0667 253.0519 223.0641	+H	0.1127
31	aristoloterpenate-I	$C_{32}H_{31}NO_8$	558.2122	10.85	558.2137	2.69	545.1974 421.1061 409.1071	+H	0.0007
32	AL-FI	$C_{16}H_{11}NO_3$	266.0811	10.98	266.0820	3.38	251.0634 195.0576 167.0596	+H	0.0277
33	Aristolochic acid II methyl ester	$C_{17}H_{11}NO_6$	326.0659	11.07	326.0658	-0.31	311.0797 283.0842 268.0620 308.0523 293.0287	+H	0.0031
34	Cepharanone-C	$C_{17}H_{11}NO_5$	310.0710	11.19	310.0715	1.61	327.0972 295.0578 267.0709	+H, +NH_4	0.0150
35	AL-II	$C_{16}H_9NO_3$	264.0655	11.22	264.0645	-3.79	236.0685 234.0803 206.0835	+H	0.0294
36	11-hydroxy-5H-[1,3]dioxolo[4',5':4,5]benzo[1,2,3-de]benzo[g]quinoline-5,6(7H)-dione	$C_{17}H_9NO_5$	308.0659	11.45	308.063 6	-7.47	278.0369 250.0510 222.0624	+H	0.0033
37	2- hydroxy -8-	$C_{17}H_{11}NO_5$	310.0710	11.75	310.0714	1.29	295.0521 280.0659 267.0469 252.0459	+H	0.0039

	methoxy- cepharanthine A								
38	aristoliukine B	$C_{17}H_{11}NO_5$	310.0710	12.00	310.0721	3.55	292.0611 278.0484 234.0559	+H	0.0179
39	AL-I	$C_{17}H_{11}NO_4$	294.0761	12.28	294.075	-3.74	279.0742 264.0411 251.0548 236.0389	+H	0.0988
40	AAII	$C_{16}H_9NO_6$	312.0503	12.30	312.0485	-5.77	294.0384 268.0592 266.0515	+H, +NH_4, +Na	5.3447
41	Aristolochic acid Ia methyl ester	$C_{17}H_{11}NO_7$	342.0608	12.31	342.0599	-2.63	364.0434 296.0683 280.0611 252.0706	Na,+ H,++ NH_4	0.0453
42	AAI	$C_{17}H_{11}NO_7$	342.0608	12.55	342.0591	-4.97	324.0498 298.0698 296.0682 281.0666 265.0362	+H, +NH_4, +Na	5.3407
43	AAIV	$C_{18}H_{13}NO_8$	372.0714	12.60	372.0715	0.27	354.0739 328.0939 326.0893 311.0835 283.0693	+H,+ Na	0.0947
44	3-hydroxy-4-methoxy-10-nitrophenanthrene-1-carboxylic acid methyl ester	$C_{17}H_{13}NO_6$	328.0815	13.01	328.0828	3.96	310.0701 295.0517 251.0607	+H	0.0056
45	aristophyllides A	$C_{44}H_{31}NO_8$	702.2122	13.56	702.2121	-0.14	516.1661 297.0829 279.0954	+H	0.0052

Table S4 Sample information of AMH

Number	Code	Content μg/g(n=2)						
		AAI	AAII	AAC	AAD	AL-I	AL-BII	AL-FI
X01	20201101	600.261	198.686	21.404	20.013	14.863	0.110	7.741
X02	20201201	533.557	88.408	17.470	2.548	3.971	0.039	2.459

X03	20200701	546.156	164.580	23.736	9.956	10.580	0.052	5.163
X04	20210326	434.861	50.795	7.620	45.294	4.680	0.021	1.275
X05	20210101	565.051	182.436	29.251	52.645	13.114	0.048	5.342
X06	20200301	481.907	99.510	12.441	59.341	6.007	0.054	2.008
X07	20201001	549.911	123.072	22.944	2.823	8.592	0.033	3.712
X08	20190601	347.624	73.910	8.104	24.115	3.646	0.029	1.352
X09	20200901	459.812	158.995	23.014	70.855	15.456	0.062	5.573
X10	20210401	513.550	140.117	20.575	20.618	8.377	0.032	4.597
X11	20210815	130.632	57.845	9.922	23.719	3.417	0.029	2.291
X12	20210829	538.644	78.446	16.641	36.010	4.914	0.048	11.280
X13	20210918	135.738	38.389	11.934	35.831	4.610	0.027	8.504
X14	20211001	369.129	136.601	12.075	23.331	4.376	0.236	22.537
X15	20211109	125.225	59.687	9.110	26.497	3.366	0.036	2.791
X16	20211121	123.657	61.920	10.766	25.173	3.501	0.035	2.612

Table S5 Sample information of ADS

Number	Code	Content μg/g(n=2)				
		AAI	AAII	AAC	AAD	AL-I
T01	20201101	10.073	8.022	1.104	120.625	15.341
T02	20200801	6.701	4.872	1.002	90.902	32.102
T03	20201001	6.924	9.243	1.241	114.870	4.861
T04	20210401	17.252	14.851	0.911	72.827	11.163
T05	20200901	6.902	4.584	1.123	94.103	20.442
T06	20201201	8.776	5.812	0.976	67.854	26.635
T07	20210101	7.866	11.123	1.889	107.247	21.610
T08	20210326	13.230	33.323	1.013	84.001	25.365
T09	20210801	12.748	5.893	0.942	67.735	1.953
T10	20210820	16.316	10.653	2.300	42.896	7.310
T11	20210901	7.985	22.767	1.273	39.960	7.034
T12	20210922	9.656	8.274	1.513	55.975	0.902

T13	20211001	12.355	6.430	1.940	58.150	8.433
T14	20211003	6.734	4.438	2.081	79.377	7.002
T15	20211118	12.049	6.032	1.616	58.052	5.774

"-",indicates not detected or below the limit of quantification.

Table S6 Sample information of ACY

Number	Code	Content μg/g(n=2)				
		AAI	AAII	AAC	AAD	AL-I
Z1	20190201	2094.5	1530.6	388.2	254.3	67.5
Z2	20190301	1780.5	1883.0	360.3	195.0	44.8
Z3	20190501	938.6	1377.6	430.2	137.8	26.0
Z4	20190601	2255.3	2118.3	130.8	108.8	26.5
Z5	20190801	1935.2	1582.8	191.7	144.9	29.4
Z6	20191001	1558.5	1394.4	273.5	234.0	33.5
Z7	20190910	2622.4	2392.3	221.5	181.4	34.9
Z8	201908	2317.6	2158.7	193.5	142.7	29.2
Z9	20190926	1797.6	1727.4	142.4	109.4	32.6
Z10	20191101	2390.1	2127.7	283.0	253.9	33.9
Z11	20191103	1286.5	2018.5	453.5	186.6	46.9
Z12	20191106	2360.4	2041.8	230.4	152.3	34.0
Z13	20191108	2405.4	2045.8	56.3	223.6	30.4
Z14	201804	2068.8	2147.0	203.8	124.4	44.0
Z15	201805	3201.5	3436.3	320.2	305.5	52.2
Z16	201811	3567.5	3679.9	527.7	206.1	52.0
Z17	201806	2109.4	1885.3	191.2	165.4	27.2
Z18	201807	2163.6	2154.5	194.6	126.1	21.0
Z19	201809	1877.7	1713.8	198.8	113.5	37.2
Z20	201901	1721.2	2182.4	300.1	147.3	51.9
Z21	201903	2800.0	3688.1	519.1	192.6	81.7

Note:This table shows the experimental data of the previous period in the laboratory

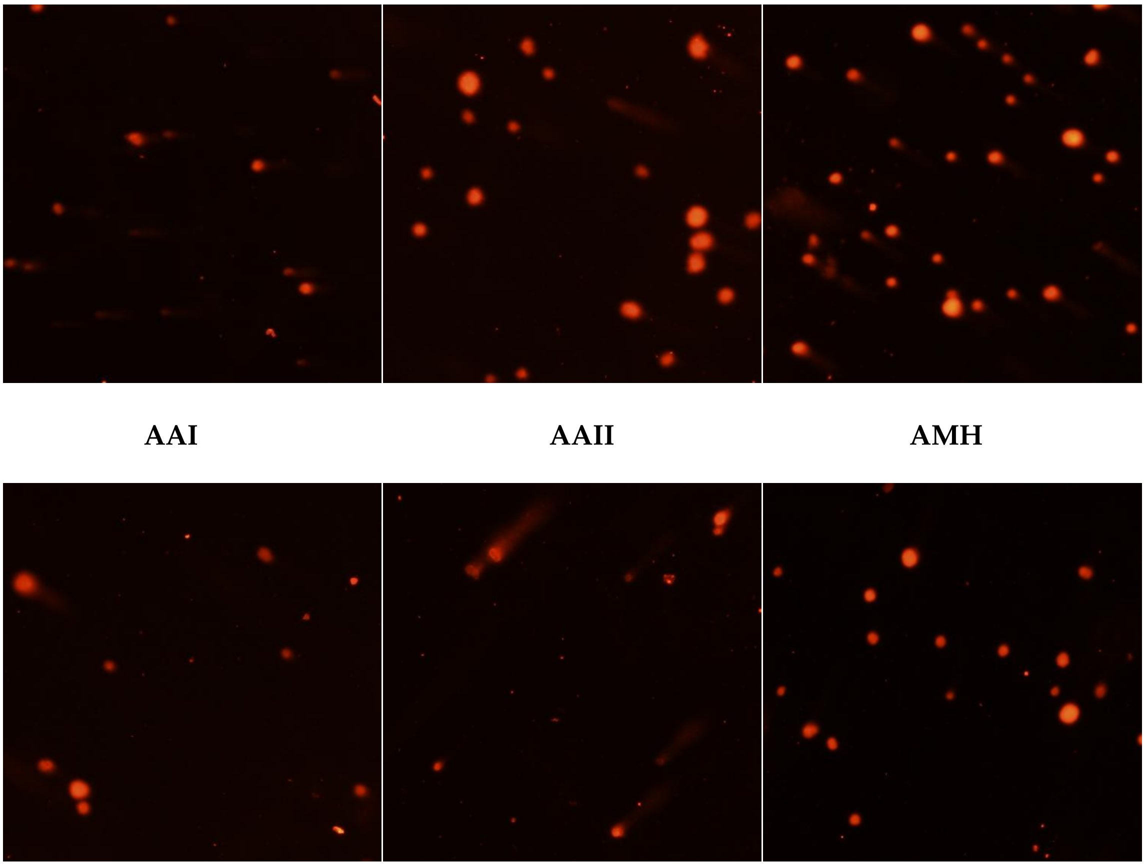

AAI AAII AMH

ADS ADM BLANK

Figure S5 Results of comet assay
